# Patient and partner experiences of prostate cancer survivorship in Ireland: findings from the co-designed PRO-ACT study (CTRIAL-IE 24–45)

**DOI:** 10.1007/s00345-026-06863-7

**Published:** 2026-11-13

**Authors:** Calvin R. Flynn, Martin Sweeney, John Sullivan, Emer Mulvaney, Jacqueline Daly, Claire Hopkins, Matthew Kiely, Vicky Donachie, Jacinta Marron, Nicola Elmer, Michael Daly, David Galvin, Ray McDermott, Anna Loughlin, Geraldine O’Boyle, Catherine McGarvey, Seamus O’Reilly, Paul J. Kelly

**Affiliations:** 1 Mater Misericordiae University Hospital, Oncology, Dublin, Ireland; 2 Royal College of Surgeons (RCSI), St Stephens Green, Dublin, Ireland; 3 Cancer Trials Ireland, Dublin, Ireland; 4 St James’s Hospital, Dublin, Ireland; 5 East Galway Midlands Cancer Support Centre, EGM, Galway, Ireland; 6 St Lukes Radiation Oncology Network, Dublin, Ireland; 7 St Vincents University Hospital, Dublin, Ireland; 8 University Hospital Galway, Galway, Ireland; 9 Bons Secours Radiotherapy UPMC, Cork, Ireland

**Keywords:** Mental health, Partner experience, Prostate cancer, Psychosexual support, Sexual wellbeing, Survivorship, Treatment preparedness

## Abstract

**Background:**

Prostate cancer (PCa) is the most commonly diagnosed malignancy among men in Ireland, yet patient and partner experiences regarding sexual wellbeing, treatment preparedness, and survivorship needs remain poorly characterised.

**Methods:**

The PRO-ACT study employed parallel, co-designed online surveys for patients with PCa and their partners across the Republic of Ireland and Northern Ireland. Surveys were developed collaboratively with a patient previously treated for PCa and disseminated online between November 2024 and June 2025. Descriptive statistics summarised demographics, treatment preparedness, partner inclusion, sexual wellbeing, mental health, multidisciplinary consultation, and urinary/bowel toxicities.

**Results:**

A total of 598 patients and 538 partners participated. Preparedness for treatment-related sexual consequences was low: 66.3% of patients felt underprepared, and 53.1% of partners received no explanation. Sexual difficulties were widespread, with 85.4% of patients reporting erectile problems and 84.3% reporting decline in sexual wellbeing; 68.0% of partners also reported deterioration. Mental health impacts were substantial (patients: 58.7%, partners: 51.5%), yet psychological support was rarely offered. Urinary incontinence (63.7%), obstructive symptoms (40.4%), and bowel disturbances (40.1%) were common. Multidisciplinary involvement was inconsistent, with only 48.6% offered cross-specialty consultations.

**Conclusions:**

This first all-island assessment demonstrates major gaps in information provision, partner inclusion, psychosexual support, and toxicity management. Findings highlight the need for structured, personalised, multidisciplinary survivorship pathways recognising sexual wellbeing and mental health as core components. Results provide actionable insights for Ireland’s next National Cancer Strategy.

**Supplementary Information:**

The online version contains supplementary material available at https://doi.org/10.1007/s00345-026-06863-7.

## Introduction

Prostate cancer (PCa) is the most commonly diagnosed malignancy among men in Ireland and across Europe [1–3]. Approximately 4,000 men are diagnosed annually in Ireland [4]. Although survival has improved with advances in detection and treatment [1–3], surgery, radiotherapy, brachytherapy, androgen deprivation therapy (ADT), and systemic therapies can result in long-term consequences extending beyond oncological outcomes [5–7]. Sexual dysfunction, urinary incontinence, and bowel toxicities are common sequelae with profound quality of life effects [5, 6]. Sexual dysfunction alone affects up to 80% of men following treatment [8], closely linked with psychological distress, relationship strain, and intimacy challenges impacting both patients and partners [9, 10].

Given these long-term effects, international guidelines from the European Association of Urology (EAU) emphasise shared decision-making, effective communication, and multidisciplinary consultation [11]. The European Society for Medical Oncology (ESMO) Expert Consensus Statements advocate coordinated, patient-centred survivorship care addressing physical, psychological, social, and sexual wellbeing [12]. Sexual wellbeing after PCa treatment is multidimensional and requires holistic care rather than purely biomedical approaches focused solely on erectile function [13, 14]. Nevertheless, patients frequently feel underinformed about treatment-related toxicities, referral pathways between specialties remain inconsistent, and access to psychosexual support and specialised rehabilitation varies considerably [15, 16].

An additional gap concerns partners, who play a central role in coping, yet their perspectives remain underrepresented in PCa research and clinical pathways. Emerging evidence indicates that partners often feel excluded from consultations, receive little information about sexual wellbeing, and experience substantial psychological burden [9, 16]. Most evidence is qualitative, with few large-scale quantitative or dyadic studies capturing both experiences in parallel. No such study has been conducted in Ireland.

To address these gaps, the Patient-led Research On Sexual Experience After Prostate Cancer Treatment (PRO-ACT) study conducted parallel surveys of patients with PCa and their partners across the Republic of Ireland (ROI) and Northern Ireland (NI). In keeping with principles of patient and public involvement (PPI) [17], PRO-ACT was co-designed by a patient with lived experience alongside patient advocates and healthcare professionals to ensure the research reflected patient priorities. The objective of PRO-ACT was to examine patient and partner perspectives on knowledge delivery, treatment preparedness, referral pathways, multidisciplinary care, partner inclusion, sexual wellbeing, mental health, and urinary and bowel toxicities. The study sought to generate a comprehensive understanding of PCa survivorship experiences across Ireland to inform survivorship care, identify research priorities, and support development of Ireland’s next National Cancer Strategy.

## Methods

### Study design and setting

This cross-sectional study employed parallel, co-designed questionnaires for patients treated for PCa and their partners across ROI and NI. Survey development was led by a patient previously treated for PCa in collaboration with a 14-member interdisciplinary steering committee comprising four patient representatives, one partner representative, and nine healthcare professionals from urology, medical oncology, radiation oncology, psychology, advanced nurse practitioners, and doctors-in-training. The patient partner had over 10 years’ experience facilitating a large PCa peer-support group and drew on insights from more than 2,000 unsolicited, confidential interactions with men affected by PCa to identify key survivorship issues and inform questionnaire development. Draft questionnaires underwent iterative review by the steering committee to ensure clinical accuracy, relevance, and patient-centred language.

Surveys were administered online via SurveyMonkey^®^ between November 2024 and June 2025, supported by Cancer Trials Ireland (CTI). Recruitment was facilitated through CTI, patient advocacy organisations, cancer support centres, hospital networks, and social media. Participation was voluntary and anonymous.

### Participants

#### Patients

Adults (≥ 18 years) residing in ROI or NI diagnosed with PCa were eligible. Patients could have received prostatectomy, external-beam radiotherapy (RT), brachytherapy, ADT, chemotherapy, or active surveillance (AS).

#### Partners

Adults (≥ 18 years) currently or previously in a relationship with a person diagnosed with PCa were eligible.

Eligibility was assessed at the consent stage using self-reported screening questions confirming age, place of residence, and relevant PCa status. Only respondents meeting the eligibility criteria and providing informed consent could proceed to the questionnaire.

### Survey content

Patient and partner questionnaires covered the following domains:

- Demographics and treatment characteristics.
- Information provision and treatment preparedness.
- Partner involvement in consultations.
- Sexual wellbeing and psychosexual support.
- Mental health and psychological care.
- Multidisciplinary care and cross-specialty referrals.
- Urinary and bowel function and toxicity management.

Survey formats included single-response, multiple-choice, and Likert-scale questions. Full questionnaires are provided in Supplemental Appendices 1 and 2.

### Data analysis

Descriptive statistics were used. Frequencies and percentages were calculated for categorical variables; means and standard deviations for continuous variables. Data were analysed using Microsoft Excel and SPSS version 28.

### Ethics

Ethical approval was obtained from the Royal College of Surgeons in Ireland (RCSI) Research Ethics Committee (REC202407054). All participants provided informed consent.

## Results

### Demographics and treatment characteristics

A total of 598 patients and 538 partners participated. Patient mean age was 65.7 years (SD 7.85). Most identified as heterosexual (88.2%). Patients predominantly resided in ROI (98.1%). The majority (73.9%) were in a sexually active relationship at the time of completing the survey. Treatment modalities were broad: 58.79% received surgery, 36.97% RT, 7.47% brachytherapy, 29.9% ADT, 4.44% chemotherapy, 10.3% AS, and 4.0% other. Partners were 84.2% female, 15.5% male, 0.3% other; mean age 64.4 years (SD 7.3). Patient and partner demographics and treatment characteristics are summarised in Table 1.

**Table 1:** Patient/partner demographics and treatment characteristics

Demographics		Patients (*N* = 598)	Partners (*N* = 538)
Age median (range)		65 (35–90)	65 (33–82)
Sex	Female	Not captured	283 (84.2%)
	Male		52 (15.5%)
	Prefer to self-describe		1 (0.3%)
Region	Republic of Ireland	490 (98.1%)	357 (99.7%)
	Northern Ireland	8 (1.6%)	1 (0.3%)
Sexual orientation	Heterosexual	412 (88.2%)	281 (87.3%)
	Homosexual	24 (5.1%)	6 (1.9%)
	Asexual	15 (3.2%)	17 (5.3%)
	Prefer not to say	12 (2.6%)	15 (4.7%)
	Bisexual	4 (0.9%)	3 (0.9%)
Education	Tertiary level	232 (46.7%)	165 (46.2%)
	Secondary level	232 (46.7%)	177 (49.6%)
	Primary level	29 (5.8%)	14 (3.9%)
	No formal education	4 (0.8%)	1 (0.3%)
Year of prostate cancer diagnosis	Pre 2006	11 (2.2%)	12 (3.6%)
	2006–2010	17 (3.4%)	21 (5.9%)
	2011–2015	60 (12.2%)	51 (14.6%)
	2016–2020	139 (28.3%)	102 (29%)
	2021-Present	260 (52.8%)	159 (45.2%)
Treatment characteristics			
Treatment modality received	Surgery	291 (58.8%)	186 (52.7%)
	Radiotherapy	183 (37.0%)	149 (42.2%)
	Hormonal therapy	148 (29.9%)	103 (29.2%)
	Active surveillance	51 (10.3%)	31 (8.8%)
	Brachytherapy	37 (7.5%)	24 (6.8%)
	Chemotherapy	22 (4.4%)	26 (7.4%)
	Focal therapy	2 (0.4%)	4 (1.1%)
	Other	20 (4.0%)	18 (5.1%)
	Unsure	7 (1.4%)	6 (1.7%)
Completed treatment	Yes	363 (74.2%)	269 (76.4%)
	No	126 (25.8%)	72 (20.5%)
	Unsure		11 (3.1%)
Duration since treatment completion median (range), years		3.0 (0–30)	3.0 (0–26)

### Information provision and treatment preparedness

Patient and partner preparedness for treatment impacts was inadequate. Regarding sexual wellbeing, 66.3% of patients felt underprepared and 25.5% completely unprepared. Only 46.9% of partners received any explanation about sexual impacts; 53.1% received none. Preparedness regarding treatment-related sexual effects is summarised in Table 2.

**Table 2:** Patient-reported preparedness and communication regarding impacts on sexual wellbeing

		Patients (*N* = 598)
Preparedness for treatment-related sexual impacts	Very prepared	38 (8.6%)
	Adequately prepared	112 (25.2%)
	Underprepared	181 (40.8%)
	Completely unprepared	113 (25.5%)
Was there any discussion of the impact of treatment on sexual function?	Yes	224 (50.9%)
	No	102 (23.2%)
	Given written information	97 (22.0%)
	Unsure	17 (3.9%)
Format of information given on the effects of treatment on sexual function	Verbal	274 (63.9%)
	Written	228 (53.2%)
	Online	37 (8.6%)
	Other	10 (2.3%)
	No information received	47 (11.0%)
How clear was the information provided?	Very clear	89 (23.8%)
	Quite clear	134 (35.8%)
	Neither clear nor unclear	101 (27.0%)
	Quite unclear	45 (12.0%)
	Very unclear	5 (1.3%)
How personalised was the information provided?	Very personalised	53 (14.2%)
	Quite personalised	75 (20.2%)
	Somewhat personalised	71 (19.1%)
	A little personalised	58 (15.6%)
	Not at all personalised	115 (30.9%)

### Partner inclusion

Partner involvement in consultations was limited. Only 65.7% of partners attended initial consultations with patients. Reported barriers to attending included being unaware of the appointment (28.6%), their partner preferring to attend alone or with someone else (23.8%), not being invited by the medical team (13.3%) and being unavailable on the day (10.5%). Partner-reported inclusion is summarised in Table 3.

**Table 3:** Partner-reported communication, inclusion, and preparedness regarding sexual wellbeing

		Partners (*N* = 538)
Present at consultation where impact on sexual function was discussed	Yes	215 (65.7%)
	No	112 (34.3%)
If present, to what extent were partners included?	Very much	49 (23.3%)
	Quite a bit	41 (19.5%)
	Somewhat	45 (21.4%)
	A little	35 (16.7%)
	Not at all	40 (19.0%)
Was partners’ sexual wellbeing discussed?	Very much	5 (1.7%)
	Quite a bit	11 (3.7%)
	Somewhat	16 (5.4%)
	A little	29 (9.9%)
	Not at all	233 (79.3%)
How personalised was the information provided?	Very personalised	24 (8.2%)
	Quite personalised	25 (8.6%)
	Somewhat personalised	30 (10.3%)
	A little personalised	59 (20.2%)
	Not at all personalised	154 (52.7%)
Did partners have adequate opportunity to ask questions about sexual wellbeing?	Yes	96 (32.9%)
	No	196 (67.1%)
Did partners receive an explanation of the sexual impacts of treatment on patients?	Yes	135 (46.9%)
	No	153 (53.1%)
Did partners feel prepared for treatment-related sexual impacts?	Very much	33 (12.0%)
	Quite a bit	34 (12.3%)
	Somewhat	70 (25.4%)
	A little	65 (23.6%)
	Not at all	74 (26.8%)
Are partners angry/frustrated about the lack of information provided on sexual health?	Very much	43 (18.7%)
	Quite a bit	40 (17.4%)
	Somewhat	49 (21.3%)
	A little	53 (23.0%)
	Not at all	45 (19.6%)

### Sexual wellbeing and psychosexual support

**Fig. 1:**
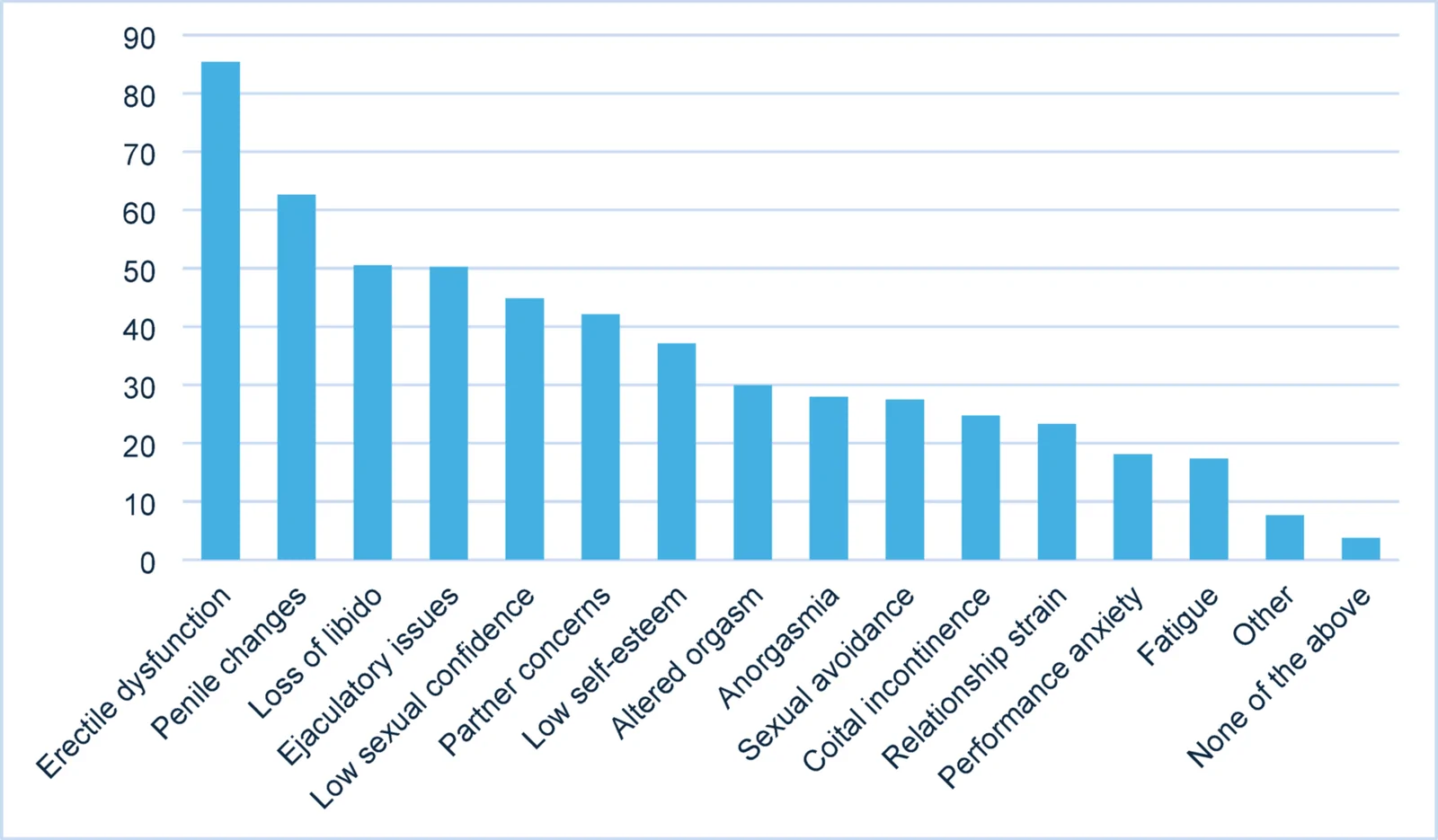
Sexual wellbeing changes reported by patients following prostate cancer treatment (%)

Sexual consequences were widespread. Among patients, 85.4% reported erectile dysfunction, 84.3% reported decline in sexual wellbeing, 50.5% loss of libido, 23.27% relationship difficulties. Among partners, 68.0% reported deterioration in their own sexual wellbeing. Patient-reported sexual wellbeing changes are summarised in Fig. 1.

Despite high prevalence, psychosexual support referral was rare. Only 11.54% of patients were offered psychosexual counselling; 4.62% attended couples counselling. Among partners, 26.3% were referred to counselling or psycho-oncology, 21.1% were directed to online resources, and 10.5% were referred to sex or couples therapy.

### Mental health and psychological support

**Fig. 2:**
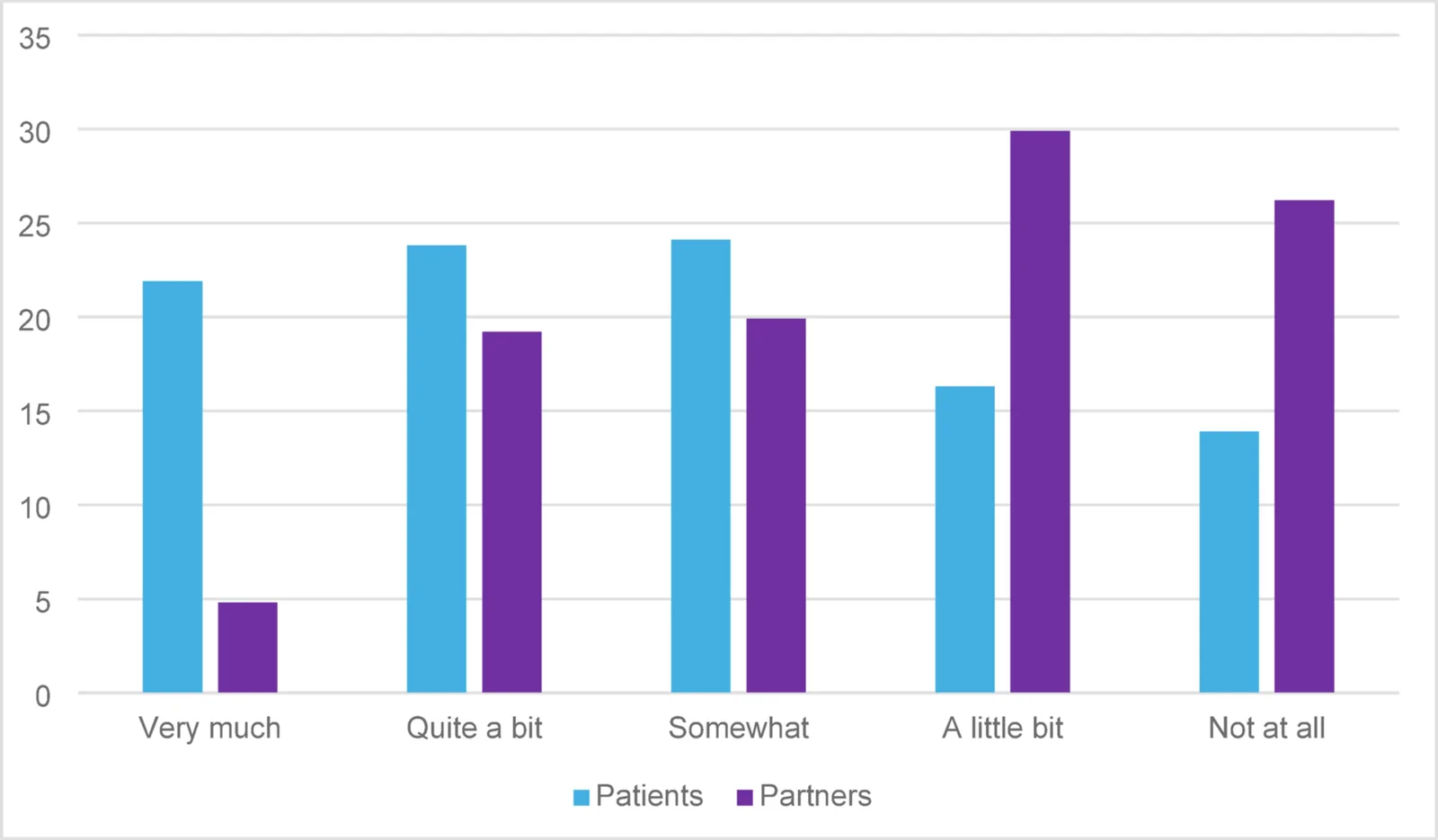
Impact of sexual wellbeing concerns on mental wellbeing in patients and partners (%)

Mental health impacts were substantial. Among patients, 58.7% reported mental health difficulties following diagnosis/treatment. Among partners, 51.5% reported mental health difficulties. Despite this, only 12.9% of patients were offered any form of hospital-based mental health support at diagnosis. Impacts of sexual wellbeing concerns on mental wellbeing for patients and partners are summarised in Fig. 2.

### Multidisciplinary care and cross-specialty referrals

Patients reported considerable variability in referral pathways during their diagnostic and treatment journey. Most consulted a urologist (92.7%), with fewer seeing a radiation oncologist (55.5%) or medical oncologist (26.6%).

Opportunities for multidisciplinary review were limited. Fewer than half of patients (48.6%) were offered consultations involving more than one specialty, and only a quarter (25.8%) were offered a second opinion within the same specialty. Over half (55.8%) did not undertake any additional consultation beyond their initial clinician interaction.

### Urinary and bowel toxicities

Urinary symptoms were common. Among patients, 63.7% experienced urinary incontinence, and 40.4% obstructive symptoms. Management was often inadequate: among those with incontinence, 34.6% received no intervention.

Bowel disturbances affected 40.1% of patients. Despite this, only 34.7% had been offered assistance, and among those who had not received support, 60.2% indicated they would have welcomed it.

## Discussion

This first comprehensive all-island assessment of PCa survivorship in Ireland demonstrates major gaps in information provision, partner inclusion, psychosexual support, and toxicity management. By combining patient and partner perspectives through co-designed surveys, PRO-ACT identifies substantial unmet survivorship needs across multiple domains. 

Preparedness for treatment consequences, particularly sexual impacts, was inadequate. Two-thirds of patients felt underprepared for sexual consequences, and over half of partners received no explanation. This parallels international findings that communication about sexual wellbeing remains insufficient [15, 16, 18]. Sexual wellbeing after PCa treatment extends beyond erectile function to encompass emotional, relational, and social dimensions [13, 14, 22–24], yet current practice appears to focus predominantly on biomedical aspects.

These deficiencies also affected partners. Although partners play central roles in treatment decision-making, emotional support, and coping within the home environment [9, 16, 18–21], opportunities for involvement during clinical consultations were limited. Many partners were not invited to consultations or survivorship discussions, restricting access to information and participation in shared decision-making within the healthcare setting. Given the influence of partner wellbeing on patient outcomes and relationship quality [9, 10, 18], greater partner inclusion should form part of routine survivorship care.

Beyond the impact on communication and relationships, psychological wellbeing represented another major unmet need. Over half of patients and partners reported mental health difficulties, yet fewer than one-sixth received support. This disconnect between need and provision is well-documented [30, 31, 32] and represents a fundamental failure to address survivorship holistically. As psychological distress and functional impairment are bidirectionally related [32], integrating mental health support into survivorship pathways is essential.

These unmet supportive care needs were accompanied by important deficiencies in care delivery. Multidisciplinary care was inconsistently delivered. Fewer than half of patients were offered cross-specialty consultations before treatment decisions, despite evidence that referral patterns and specialty of consultation influence treatment selection [27, 28, 29]. Limited multidisciplinary involvement may restrict informed decision-making and contribute to decision regret [26], highlighting incomplete implementation of current guideline recommendations [11, 12].

In addition to gaps in decision-making pathways, management of treatment-related toxicities requires improvement. Urinary and bowel toxicities were common, yet many patients received no intervention despite the substantial effects of these complications on quality of life and psychological wellbeing [5, 6, 25, 32]. More proactive, specialist-led survivorship services are needed.

Collectively, these findings align with emerging European evidence highlighting unmet patient and partner needs in PCa survivorship [18]. The ESMO survivorship framework emphasises coordinated, personalised approaches addressing physical, psychological, and social wellbeing [12], while the NHS stratified pathways model [33] provides a potential framework for implementation within Ireland.

A major strength of PRO-ACT is its co-designed methodology, ensuring that study priorities reflected lived experience. The large sample size, nationwide recruitment, parallel patient-partner design, and anonymous survey format strengthen the findings, while detailed mapping of referral pathways and information provision provides actionable evidence for service improvement.

These findings should, however, be interpreted in light of several limitations. These include the cross-sectional design, self-reported outcomes, and potential selection bias associated with online recruitment through advocacy networks and social media. The online format may also have underrepresented individuals with limited digital literacy. Cancer stage and risk stratification were not collected, treatment details could not be clinically verified, and only eight patient respondents were from NI, limiting regional comparisons. Finally, the descriptive design precluded comparisons between treatment groups.

Despite these limitations, the findings provide actionable insights for Ireland’s next National Cancer Strategy to help define the umbrella care needs of patients and their partners [34]. Key recommendations include: firstly structured information provision addressing sexual, urinary, bowel, and mental health impacts with partner inclusion; secondly routine psychosexual support and couple’s counselling integration; thirdly psychological care as standard survivorship component; fourthly mandatory multidisciplinary consultations before treatment decisions; finally specialist-led toxicity management pathways; and the development of stratified survivorship care models.

## Conclusions

This first comprehensive all-island assessment of PCa survivorship in Ireland demonstrates substantial unmet needs in information provision, partner inclusion, psychosexual support, psychological care, and toxicity management. Sexual wellbeing and mental health should be recognised as core components of survivorship care. These findings support the development of structured, multidisciplinary survivorship pathways and provide an evidence base to inform Ireland’s next National Cancer Strategy.

## Electronic Supplementary Material

Below is the link to the electronic supplementary material.

Partner Survey

Patient Survey

## Data Availability

No datasets were generated or analysed during the current study.
