# Supplementary material for: Patient and partner experiences of prostate cancer survivorship in Ireland: findings from the co-designed PRO-ACT study (CTRIAL-IE 24–45)
Source: World J Urol. 2026 Nov 13;44(1):805. doi: 10.1007/s00345-026-06863-7 (PMC13768403; doi:10.1007/s00345-026-06863-7)
Supplement: Supplementary file 1 [file 345_2026_6863_MOESM1_ESM.pdf]

Cancer Trials Ireland CLG
RCSI House
121 St Stephen's Green
Dublin 2
D02 H903
Ireland
e: info@cancertrials.ie
t: +353 (0)1 6677211
f: +353 (0)1 6697869

Together, we're finding answers to cancer.

CTRIAL-IE 24-45 PRO-ACT: Patient-led Research On sexual experience After prostate Cancer Treatment

Survey Questionnaire

Dear Participant,

What is the study about?

You are invited to take part in a research study in the form of an anonymous survey which aims to better understand the sexual experience of patients and partners after prostate cancer treatment. The survey includes questions about your sexual wellbeing and mental health. We will also ask you about the information you received about the possible sexual side effects of prostate cancer treatment and about supports you may have been offered. We appreciate, that for some men, sexual health prior to a prostate cancer diagnosis may not have been optimal as erectile dysfunction may also precede treatment for some men. The survey includes questions which you may find uncomfortable reading or answering, overly personal or possibly distressing about sexual practices, drug use, mental health. You do not have to complete the survey all in one go – you can take a break and come back to it if you need to. All questions are optional and all answers will be collected anonymously - you will not be able to be identified. You can also choose not to take part at all.

The study has been developed by a patient previously treated for prostate cancer and a partner of a patient and a cohort of patients with the same disease. The interdisciplinary steering committee have also helped to design the study and is comprised of Urologists, Medical Oncologists, Radiation Oncologists, Advanced Nurse Practitioners specialising in Prostate Cancer, doctors in training, Psychologists, patient and partner representatives. The co Chief – Investigators of this study are Mr. Martin Sweeney (Patient Consultant Committee, Cancer Trials Ireland) and Dr. Paul Kelly (Radiation Oncologist, UPMC Bons Secours Radiotherapy Cork and Genitourinary Disease-specific subgroup chair Cancer Trials Ireland). This study is being conducted with Cancer Trials Ireland (http://www.cancertrials.ie) as part of their public patient involvement (PPI) initiative in cancer clinical research. This partnership will increase the reach of the study and facilitate analysis of the data received and dissemination of the findings. We plan for a total of 300 patients treated for prostate cancer and 300 partners of patients treated for prostate cancer to complete the survey so that we gather information on a broad range of experiences across Ireland and Northern Ireland.

What will I have to do?

Ms Deirdre Somers (Chairperson), Prof. Seamus O'Reilly (Clinical Lead), Prof. Ray McDermott (Vice Clinical Lead), Ms Anne Byrne, Mr Michael Daughton, Mr Paul Egan, Prof. John Kennedy, Mr Patrick Kivlehan, Ms Sharen McCabe, Mr Rory Montgomery, Prof. Leonie Young. Ms Eibhlin Mulroe (CEO), Ms Angela Clayton-Lea (COO), Mr David Donovan (Company Secretary).
Cancer Trials Ireland CLG registered in Dublin, Ireland. Registered Office: Cancer Trials Ireland, RCSI House, 121 St Stephen's Green, Dublin 2, D02 H903, Ireland
Charity Regulatory Authority No. 20036676 | Revenue No. CHY12492 | Company No. 268044

As part of this study, you will be required to answer questions in an anonymous online survey relating to your experiences as a partner of a patient having and being treated for prostate cancer and your experience after treatment, in particular in relation to sexual wellbeing. As a part of the survey we would like to ask your opinion on the topics of consent and Data Protection (GDPR) in clinical trials. These questions are not specifically related to the topic of this survey but relate more broadly to clinical research. All survey questions are optional. Your full participation will take approximately 15 - 20 minutes (which includes reading this information sheet, reading and filling in the questionnaire).

What are the benefits?

The study's findings will help improve and understand further the impact that sexual wellbeing after treatment for prostate cancer has on the lives of patients and their partners in Ireland and Northern Ireland and their partners. It will investigate information and communication needs of patients and their partners. We hope that the results of this survey will be used to guide evidence-based changes in service provision and to also guide the implementation of new health and social care resources to optimise care for patients with prostate cancer with particular emphasis on sexual wellbeing and mental health after treatment.

What are the risks?

As the survey contains questions of a personal nature reading and answering the questions may lead to increased anxiety. Sexual side effects can be a sensitive subject and you may find this survey intrusive or distressing. It may also raise questions or concerns about your partner’s treatment and where you can find information and support to deal with side effects. The survey may include questions about sexual practices, drug use, mental health which you may find distressing or overly personal. All questions are optional and all answers will be collected anonymously, you will not be able to be identified. If you feel upset, distressed, or uncomfortable, support is available from cancer support groups and charities. Contact details and further information can be found via this HSE link: -
https://www.hse.ie/eng/services/list/5/cancer/patient/patlinks/

What if I do not want to take part?

Participation in this study is voluntary, and you can choose not to take part. It is entirely up to you.

What happens to the information? Will my identity and my answers to the survey be kept private?

Your responses will be completely anonymous and confidential. We will not collect any identifying information such as your name, email address or electronic IP address. Your browser may store Survey Monkey cookies to allow you to resume a partially completed survey. Cancer Trials Ireland will not have access to any information other than actual question responses. You will not be identified in any reports

Cancer Trials Ireland CLG
RCSI House
121 St Stephen's Green
Dublin 2
D02 H903
Ireland
e: info@cancertrials.ie
t: +353 (0)1 6677211
f: +353 (0)1 6697869

or publications. However, due to the anonymous nature of the survey, there will be no way to withdraw responses once you have submitted them.

The collected information will be kept private and stored securely and safely on a SurveyMonkey® single password protected account managed by Cancer Trials Ireland. As you will not be asked to provide any identifying details, the data you provide will be anonymous. At regular intervals during the survey and following completion of the study the anonymous data will be downloaded from the SurveyMonkey® platform and saved as a database on a secure server at Cancer Trials Ireland. The anonymous data gathered in the study will be kept for ten years.

Who can take part?

You can take part if you are aged 18 years or older and are a partner of someone who has been diagnosed with prostate cancer and are able to complete the online questionnaire independently or with the help of a friend or relative.

What happens at the end of the study?

The information you provide will be pooled together with the information completed by other survey participants and the results may contribute to research publications and/or conference presentations. As detailed above, because no personal identifiable information will be collected, the information published will never be linked with any individual person. If you choose to add free text comments to survey answers, these may be used anonymously as a part of the final trial report or in presentations. You will not be identified.

What if I have more questions or need help understanding something?

If you have any questions or need help understanding something before deciding whether or not to take part, please feel free to contact the researchers Mr Martin Sweeney or Dr Paul Kelly via: proact@cancertrials.ie

This study has obtained ethical approval from the RCSI Research Ethics Committee.

Thank you for taking the time to read this information. We would be grateful if you participated in this study.

Cancer Trials Ireland CLG
RCSI House
121 St Stephen's Green
Dublin 2
D02 H903
Ireland
e: info@cancertrials.ie
t: +353 (0)1 6677211
f: +353 (0)1 6697869

Together, we're finding answers to cancer.

Consent and Eligibility

**Do you consent to participate in this study?**	**Yes**		**No**	

**By ticking this box, I agree to an anonymised report of this survey being published**	

**Please confirm that you are a partner of a patient in Ireland and Northern Ireland who has been diagnosed with prostate cancer.**	**Yes**		**No**	

**Please indicate how you will complete the survey?**	
• **Independently**	
• **With the help of a relative / friend**	
• **A combination of independently and with the help of a relative / friend**	

Cancer Trials Ireland CLG
RCSI House
121 St Stephen's Green
Dublin 2
D02 H903
Ireland
e: info@cancertrials.ie
t: +353 (0)1 6677211
f: +353 (0)1 6697869

Together, we're finding answers to cancer.

Demographics

**1. Your Age**	

**2. Year of partner's prostate cancer diagnosis– please provide best estimate**	

**3. What is the highest level of education you have attained?**	
• No formal education	
• Primary school	
• Secondary school	
• Third level	

**4. County of Residence**							
Antrim		Armagh		Carlow		Cavan	
Clare		Cork		Derry		Donegal	
Down		Dublin		Fermanagh		Galway	
Kerry		Kildare		Kilkenny		Laois	
Leitrim		Limerick		Longford		Louth	
Mayo		Meath		Monaghan		Offaly	
Roscommon		Sligo		Tipperary		Tyrone	
Waterford		Westmeath		Wexford		Wicklow	

**5. What treatment did your partner have for prostate cancer?** *(please tick all that apply)*	
• Surgery (Radical Prostatectomy)	
• Radiotherapy	
• Brachytherapy	
• Hormone treatment (injections / tablets)	
• Chemotherapy	
• Focal therapy (Cryotherapy / High-intensity focused ultrasound)	
• Active surveillance or watchful waiting	
• I don't know / unsure	
• Other	
If other, please specify	

Cancer Trials Ireland CLG
RCSI House
121 St Stephen's Green
Dublin 2
D02 H903
Ireland
e: info@cancertrials.ie
t: +353 (0)1 6677211
f: +353 (0)1 6697869

**6. Has your partner finished their treatment?**	**Yes**		**No**	
**If yes, how long has it been since your partner's treatment finished? (Your best estimate)**	**Years and Months**			
**If No, what treatment is your partner currently receiving?**				
• Surgery (Radical prostatectomy)				
• Radiotherapy				
• Brachytherapy				
• Hormone treatment (injections / tablets)				
• Chemotherapy				
• Focal therapy (Cryotherapy / High-intensity focused ultrasound)				
• Active surveillance or watchful waiting				
• I don't know				
• Other				

**7. Gender: How do you identify?**	
• Female	
• Male	
• Non-binary	
• Prefer to self-describe	

**8. Which of the following best represents your sexual orientation?**	
• Asexual	
• Bisexual	
• Gay	
• Heterosexual or straight	
• Lesbian	
• Pansexual	
• Prefer not to say	

**9. Do you consider yourself to be transgender?**	**Yes**		**No**	

Cancer Trials Ireland CLG
RCSI House
121 St Stephen's Green
Dublin 2
D02 H903
Ireland
e: info@cancertrials.ie
t: +353 (0)1 6677211
f: +353 (0)1 6697869

**Understanding and knowledge of the impact of prostate cancer treatment on sexual wellbeing.** *(For any question where you can type a response, please take care not to include any person's name or personal information for anonymity and privacy reasons)*		
**10. Were you present at an appointment where the impact on sexual function was discussed with your partner?**	**Yes**	**No**
**If no, why were you not present at such an appointment**		
• I was unavailable / unable to attend		
• I was unaware of the appointment but learnt of it later		
• My partner preferred to go alone / with another support person / I was not asked to attend		
• The medical team did not invite me into the room for the consultation		
• Prefer not to say		
• I am unaware if such an appointment / discussion occured		
• Other		
If other, please specify		
**If yes, to what extent were you included in the conversation**		
• Very much		
• Quite a bit		
• Somewhat		
• A little		
• Not at all		

**11. Was your <u>own sexual wellbeing mentioned or discussed with you</u> at any stage throughout your partner's prostate cancer diagnosis and / or treatment?**	
• Very much	
• Quite a bit	
• Somewhat	
• A little	
• Not at all	

**12. How personalised / tailored to you and your partner's situation / circumstances was the information provided throughout your partner's diagnosis and treatment?**	
• Very personalised / tailored to me and my partner's situation	
• Quite personalised / tailored to me and my partner's situation	

Cancer Trials Ireland CLG
RCSI House
121 St Stephen's Green
Dublin 2
D02 H903
Ireland
e: info@cancertrials.ie
t: +353 (0)1 6677211
f: +353 (0)1 6697869

• Somewhat personalised / tailored to me and my partner's situation	
• A little personalised / tailored to me and my partner's situation	
• Not at all personalised / tailored to me and my partner's situation	

**13. How much did the healthcare system include <u>you as the partner</u> in conversations about sexual wellbeing ?**	
• Very much	
• Quite a bit	
• Somewhat	
• A little	
• Not at all	

**14. Did you have adequate opportunity to ask questions during conversations about sexual wellbeing throughout your partner's prostate cancer diagnosis and / or treatment?**	**Yes**	**No**

**15. Were there important questions that you wanted to ask the medical team throughout your partner's cancer diagnosis and treatment but didn't?**	**Yes**	**No**
**If yes, why did you not ask these questions?** *(Please tick all that apply)*		
• I was reluctant / did not wish to discuss the issues with my partner / family member present		
• I did not feel it was important to me		
• I was embarrassed / uncomfortable discussing sexual wellbeing		
• The topic did not come up / Doctor did not bring it up or the doctor / scenario did not invite questions		
• There wasn't enough time at any appointments I attended to ask questions		
• I was overwhelmed / distressed by my partner's cancer diagnosis and did not consider beyond treatment		
• I did not feel my partner would want me to		
• I did not go to any appointments with my partner		
• Other		
If other, please specify		

Cancer Trials Ireland CLG
RCSI House
121 St Stephen's Green
Dublin 2
D02 H903
Ireland
e: info@cancertrials.ie
t: +353 (0)1 6677211
f: +353 (0)1 6697869

Sexual counselling – Information provided and its efficacy.

(For any question where you can type a response, please take care not to include any person's name or personal information for anonymity and privacy reasons)

**16. Did you receive an explanation of the sexual side effects of prostate cancer procedures and/or treatments for your partner**	**Yes**	**No**
**If yes, was this explanation satisfactory?.**		
• Very much		
• Quite a bit		
• Somewhat		
• A little bit		
• Not at all		

**17. Did you receive enough information about sexual health after prostate cancer?**	**Yes**	**No**
**If no, are you frustrated/angry about the lack of information you received about sexual health after prostate cancer.**		
• Very much		
• Quite a bit		
• Somewhat		
• A little bit		
• Not at all		

**18. I felt prepared for the sexual side effects**	
• Very much	
• Quite a bit	
• Somewhat	
• A little bit	
• Not at all	

**19. <u>Today</u>, how would you rate the impact of your partner's treatment on your sexual wellbeing based on what your expected?**<br><br>**The impact has been:**	
• Much better than I expected	
• Better than I expected	
• What I expected	
• Worse than I expected	
• Much worse than I expected	

Cancer Trials Ireland CLG
RCSI House
121 St Stephen's Green
Dublin 2
D02 H903
Ireland
e: info@cancertrials.ie
t: +353 (0)1 6677211
f: +353 (0)1 6697869

Mental Health

(For any question where you can type a response, please take care not to include any person's name or personal information for anonymity and privacy reasons)

**20. Has your mental health been impacted since your partner's prostate cancer diagnosis and / or treatment?**	**Yes**		**No**	

**21. To what extent have sexual wellbeing concerns or sexual function impacted your mental wellbeing**	
• Very much	
• Quite a bit	
• Somewhat	
• A little bit	
• Not at all	

Cancer Trials Ireland CLG
RCSI House
121 St Stephen's Green
Dublin 2
D02 H903
Ireland
e: info@cancertrials.ie
t: +353 (0)1 6677211
f: +353 (0)1 6697869

Sexual Wellbeing

(For any question where you can type a response, please take care not to include any person's name or personal information for anonymity and privacy reasons)

**22. How important was sexual activity to you before your partner's cancer diagnosis?**	
• Very important	
• Fairly important	
• Neither important nor unimportant	
• Fairly unimportant	
• Very unimportant	

**23. How important is sexual activity to you now?**	
• Very important	
• Fairly important	
• Neither important nor unimportant	
• Fairly unimportant	
• Very unimportant	

**24. How would you rate your <u>current</u> satisfaction with your sexual wellbeing?**	
• **Very satisfied**	
• **Satisfied**	
• **Neutral**	
• **Dissatisfied**	
• **Very dissatisfied**	

**25. Since your partner's treatment, have you experienced changes to your feeling of sexual wellbeing and satisfaction?**	**Yes**		**No**	
**(If yes), How much change have you experienced to your feeling of sexual wellbeing and satisfaction?**				
• **Very big change**				
• **Big change**				
• **Moderate change**				
• **Small change**				
• **Very small change**				
**If yes, have you sought help or support**	**Yes**		**No**	
**If no, why did you not seek help or support?** *(Please tick all that apply)*				
• I was reluctant / did not wish to discuss the issues with my partner / family member present				

Cancer Trials Ireland CLG
RCSI House
121 St Stephen's Green
Dublin 2
D02 H903
Ireland
e: info@cancertrials.ie
t: +353 (0)1 6677211
f: +353 (0)1 6697869

• I did not feel it was important to me	
• I was embarrassed / uncomfortable discussing sexual wellbeing	
• The topic did not come up / Doctor did not bring it up or the doctor / scenario did not invite questions	
• There wasn't enough time at any appointments I attended to ask questions	
• I was overwhelmed / distressed by my partner's cancer diagnosis and did not consider beyond treatment	
• Other	
If other, please specify	
**If you have sought help or support in relation to changes to your feeling of sexual wellbeing and satisfaction, what supports were offered** *(Please tick all that apply)*	
• Counselling /Psycho-Oncology / Psychologist	
• Referral to sex / couples therapy	
• Online / website links	
• Supports were offered to my partner – medications / erectile dysfunction clinic / vacuum pump clinic / referral to a specialist service	
• No supports were offered	
• Other	
If other, please specify	
**If supports were offered, how satisfied were you with the supports offered**	
• Very satisfied	
• Satisfied	
• Neutral	
• Dissatisfied	
• Very dissatisfied	

Cancer Trials Ireland CLG
RCSI House
121 St Stephen's Green
Dublin 2
D02 H903
Ireland
e: info@cancertrials.ie
t: +353 (0)1 6677211
f: +353 (0)1 6697869

**26. Since treatment, has your partner experienced urinary incontinence?**	**Yes**	**No**
**If yes, how much of a problem is this for you today?**		
• A big problem		
• A moderate problem		
• A small problem		
• A very small problem		
• No problem		
**If yes, to what extent do you feel that your sex life has been affected by your partner's urinary incontinence symptoms?**		
• A big problem		
• A moderate problem		
• A small problem		
• A very small problem		
• No problem		

**27. Since treatment, has your partner experienced obstructive urinary symptoms – difficulty passing water, painful urination, poor flow, urinating a lot at night etc?**	**Yes**	**No**
**If yes, how much of a problem is this for you today?**		
• A big problem		
• A moderate problem		
• A small problem		
• A very small problem		
• No problem		
**If yes, to what extent do you feel that your sex life has been affected by your partner's obstructive urinary symptoms?**		
• A big problem		
• A moderate problem		
• A small problem		
• A very small problem		
• No problem		

Cancer Trials Ireland CLG
RCSI House
121 St Stephen's Green
Dublin 2
D02 H903
Ireland
e: info@cancertrials.ie
t: +353 (0)1 6677211
f: +353 (0)1 6697869

**28. Since treatment, has your partner experienced bowel symptoms – pain, passing more wind than before, leakage from back passage, diarrhoea, constipation, bleeding etc?**	**Yes**		**No**	
**If yes, how much of a problem is this for you today?**				
• A big problem				
• A moderate problem				
• A small problem				
• A very small problem				
• No problem				
**If yes, to what extent do you feel that your sex life has been affected by your partner's bowel symptoms?**				
• A big problem				
• A moderate problem				
• A small problem				
• A very small problem				
• No problem				

**29. Have you used any of the following to improve sexual pleasure post treatment?** *(please tick all that apply)*	
• Sex toys or aids	
• Visual aids, e.g. pornography	
• Lubricants	
• Amyl-nitrate / recreational drugs	
• Vacuum devices	
• Exploring other non-penetrative ways of pleasure	
• None of the above	
• Other	
If other, please specify	
**If you have used any of the above to improve sexual pleasure, did you find it helped?**	
• Extremely helpful	
• Very helpful	
• Somewhat helpful	
• Not very helpful	
• Not at all helpful	

Cancer Trials Ireland CLG
RCSI House
121 St Stephen's Green
Dublin 2
D02 H903
Ireland
e: info@cancertrials.ie
t: +353 (0)1 6677211
f: +353 (0)1 6697869

Together, we're finding answers to cancer.

**30. Are you comfortable trying new ways to achieve sexual satisfaction outside of penetrative intercourse?**	
• Very much	
• Quite a bit	
• Somewhat	
• A little	
• Not at all	
**31. Are you content with hugging, kissing and other intimate activities, rather than penetrative sexual intercourse?**	
• Very much	
• Quite a bit	
• Somewhat	
• A little	
• Not at all	

**32. Do you talk openly with your partner about the changes to your sex life after prostate cancer treatment?**	
• Yes	
• No	
• Not applicable – there have been no changes	
**If No, why would you say this is?** *(Please tick all that apply)*	
• I am embarrassed / uncomfortable discussing the topic	
• I did not feel it was important to me / it's not relevant to us	
• The topic did not come up / I am unsure how to talk about it	
• I do not want to distress / upset my partner	
• Prefer not to say	
• **Other**	
If other, please specify	

**33. Has your connection as a couple been impacted by your partner's prostate cancer diagnosis and treatment?**	
• Strongly improved	
• Somewhat improved	
• Neither improved nor disimproved	
• Somewhat disimproved	
• Strongly disimproved	

cancer trials ireland
Together, we're finding answers to cancer.

Cancer Trials Ireland CLG
RCSI House
121 St Stephen's Green
Dublin 2
D02 H903
Ireland
e: info@cancertrials.ie
t: +353 (0)1 6677211
f: +353 (0)1 6697869

**34. What do you miss most about sex before your partner's treatment?** *(Please tick all that apply)*	
• Having a happy sex life	
• Spontaneity	
• An erection sufficient for penetrative intercourse (without using rehabilitation methods)	
• My partner's sexual self-confidence	
• My own sexual self-confidence	
• Sense of overall sexual pleasure	
• My partner's pre-treatment sex drive	
• Feeling sexually attractive to your partner	
• All of the above	
• Some change but I don't miss anything	
• Not applicable/No change, everything is the same/None of the above.	
• Other	
If other, please specify	

**Of these, which THREE do you miss the most?** *(Please select the top 3 things you miss the most)*	
• Having a happy sex life	
• Spontaneity	
• An erection sufficient for penetrative intercourse (without using rehabilitation methods)	
• My partner's Sexual self-confidence	
• My own sexual self-confidence	
• Sense of overall sexual pleasure	
• My partner's pre-treatment sex drive	
• Feeling sexually attractive to your partner	
• Some change but I don't miss anything	
• Not applicable/No change, everything is the same/None of the above.	
• Other	
If other, please specify	

Cancer Trials Ireland CLG
RCSI House
121 St Stephen's Green
Dublin 2
D02 H903
Ireland
e: info@cancertrials.ie
t: +353 (0)1 6677211
f: +353 (0)1 6697869

In the final section of this survey, we would like to ask your opinion on the topics of consent and Data Protection (GDPR) in clinical trials. These questions are not specifically related to the topic of this survey but relate more broadly to clinical research.

35. Consider the following scenario – you have been diagnosed with lung cancer, and your treating doctor has recommended that you participate in a clinical trial. After having all aspects of the trial and treatment explained to you by your medical team, you agree (consent) to take part in the trial.

In this scenario, if all your identifiable information was removed, would you be willing to extend consent to include use of your health information/samples for existing, or future research projects in any form of cancer?

• Yes	
• No	
• Unsure	

36. The General Data Protection Regulation (GDPR) is an EU law that protects personal data and privacy for individuals within the EU, including Ireland. Under GDPR, data must be processed lawfully, fairly, and transparently, collected for specific purposes, minimized, accurate, stored only as necessary, and secured. Irish individuals have rights to access, rectify, erase, restrict processing, data portability, and object to data processing, including automated decision-making. Amongst other things, organizations in Ireland must ensure explicit consent for processing sensitive data.

Should the Government simplify how GDPR is applied in some areas and remove some restrictions in relation to use of health information for research purposes?

• Strongly agree	
• Agree	
• Neither agree nor disagree	
• Disagree	
• Strongly disagree	
