# Supplementary material for: Patient and partner experiences of prostate cancer survivorship in Ireland: findings from the co-designed PRO-ACT study (CTRIAL-IE 24–45)
Source: World J Urol. 2026 Nov 13;44(1):805. doi: 10.1007/s00345-026-06863-7 (PMC13768403; doi:10.1007/s00345-026-06863-7)
Supplement: Supplementary file 2 [file 345_2026_6863_MOESM2_ESM.pdf]

Cancer Trials Ireland CLG
RCSI House
121 St Stephen's Green
Dublin 2
D02 H903
Ireland
e: info@cancertrials.ie
t: +353 (0)1 6677211
f: +353 (0)1 6697869

CTRIAL-IE 24-45 PRO-ACT: Patient-led Research On sexual experience After prostate Cancer Treatment

Survey Questionnaire

Dear Participant,

What is the study about?

You are invited to take part in a research study in the form of an anonymous survey which aims to better understand the sexual experience of patients and partners after prostate cancer treatment. The survey includes questions about your sexual function, sexual wellbeing and mental health. We will also ask you about the information you received about the possible sexual side effects of prostate cancer treatment and about supports you may have been offered. We appreciate, that for some men, sexual health prior to a prostate cancer diagnosis may not have been optimal as erectile dysfunction may also precede treatment for some men. The survey includes questions which you may find uncomfortable reading or answering, overly personal or possibly distressing about sexual practices, drug use, mental health. You do not have to complete the survey all in one go – you can take a break and come back to it if you need to. All questions are optional and all answers will be collected anonymously - you will not be able to be identified. You can also choose not to take part at all.

The study has been developed by a patient previously treated for prostate cancer and a partner of a patient and a cohort of patients with the same disease. The interdisciplinary steering committee have also helped to design the study and is comprised of Urologists, Medical Oncologists, Radiation Oncologists, Advanced Nurse Practitioners specialising in Prostate Cancer, doctors in training, Psychologists, patient and partner representatives. The co-Chief-Investigators of this study are Mr Martin Sweeney (Patient Consultant Committee, Cancer Trials Ireland) and Dr Paul Kelly (Radiation Oncologist, UPMC Bon Secours Radiotherapy Cork and Genitourinary Disease-specific subgroup chair, Cancer Trials Ireland). This study is being conducted with Cancer Trials Ireland (http://www.cancertrials.ie) as part of their public patient involvement (PPI) initiative in cancer clinical research. This partnership will increase the reach of the study and facilitate analysis of the data received and dissemination of the findings. We plan for a total of 300 patients treated for prostate cancer and 300 partners of patients treated for prostate cancer to complete the survey so that we gather information on a broad range of experiences across Ireland and Northern Ireland.

What will I have to do?

As part of this study, you will be asked to answer questions in an anonymous online survey relating to your experiences of having and being treated for prostate cancer and your experience after treatment, in particular in relation to sexual wellbeing. As a part of the survey we would like to ask your opinion on

Ms Deirdre Somers (Chairperson), Prof. Seamus O'Reilly (Clinical Lead), Prof. Ray McDermott (Vice Clinical Lead), Ms Anne Byrne, Mr Michael Daughton, Mr Paul Egan, Prof. John Kennedy, Mr Patrick Kivlehan, Ms Sharen McCabe, Mr Rory Montgomery, Prof. Leonie Young. Ms Eibhlin Mulroe (CEO), Ms Angela Clayton-Lea (COO), Mr David Donovan (Company Secretary).
Cancer Trials Ireland CLG registered in Dublin, Ireland. Registered Office: Cancer Trials Ireland, RCSI House, 121 St Stephen's Green, Dublin 2, D02 H903, Ireland
Charity Regulatory Authority No. 20036676 | Revenue No. CHY12492 | Company No. 268044

the topics of consent and Data Protection (GDPR) in clinical trials. These questions are not specifically related to the topic of this survey but relate more broadly to clinical research. All survey questions are optional. Your full participation will take approximately 20-25minutes (which includes reading this information sheet, reading and filling in the questionnaire).

What are the benefits?

The study's findings will help improve and understand further the impact that sexual wellbeing has on the lives of patients and their partners in Ireland and Northern Ireland after treatment for prostate cancer. It will investigate information and communication needs of patients and their partners. We hope that the results of this survey will be used to guide evidence-based changes in service provision and to also guide the implementation of new health and social care resources to optimise care for patients with prostate cancer with particular emphasis on sexual wellbeing and mental health after treatment.

What are the risks?

As the survey contains questions of a personal nature reading and answering the questions may lead to increased anxiety. Sexual side effects can be a sensitive subject and you may find this survey intrusive or distressing. It may also raise questions or concerns about your treatment and where you can find information and support to deal with side effects. The survey may include questions about sexual practices, drug use, mental health which you may find distressing or overly personal. All questions are optional and all answers will be collected anonymously, you will not be able to be identified. If you feel upset, distressed, or uncomfortable, support is available from cancer support groups and charities. Contact details and further information can be found via this HSE link: - https://www.hse.ie/eng/services/list/5/cancer/patient/patlinks/

What if I do not want to take part?

Participation in this study is voluntary, and you can choose not to take part. It is entirely up to you.

What happens to the information? Will my identity and my answers to the survey be kept private?

Your responses will be completely anonymous and confidential. We will not collect any identifying information such as your name, email address or electronic IP address. Your browser may store Survey Monkey cookies to allow you to resume a partially completed survey. Cancer Trials Ireland will not have access to any information other than actual question responses. You will not be identified in any reports or publications. However, due to the anonymous nature of the survey, there will be no way to withdraw responses once you have submitted them.

The collected information will be kept private and stored securely and safely on a SurveyMonkey® single password protected account managed by Cancer Trials Ireland. As you will not be asked to provide any identifying details, the data you provide will be anonymous. At regular intervals during the survey, and following completion of the study the anonymous data will be downloaded from the SurveyMonkey® platform and saved as a database on a secure server at Cancer Trials Ireland. The anonymous data

gathered in the study will be kept for ten years.

Who can take part?

You can take part if you are aged 18 years or older and have been diagnosed with prostate cancer. You must have undergone surgery or radiation or drug treatment, either on their own or in combination to treat your prostate cancer. You must be able to complete the online questionnaire independently or with the help of a friend or relative.

What happens at the end of the study?

The information you provide will be pooled together with the information completed by other survey participants and the results may contribute to research publications and/or conference presentations. As detailed above, because no personal identifiable information will be collected, the information published will never be linked with any individual person. If you choose to add free text comments to survey answers, these may be used anonymously as a part of the final trial report or in presentations. You will not be identified.

What if I have more questions or need help understanding something?

If you have any questions or need help understanding something before deciding whether or not to take part, please feel free to contact the researchers Mr Martin Sweeney or Dr Paul Kelly via: proact@cancertrials.ie

This study has obtained ethical approval from the RCSI Research Ethics Committee.

Thank you for taking the time to read this information. We would be very grateful if you participated in this study.

Together, we're finding answers to cancer.

Consent and Eligibility

**Do you consent to participate in this study?**	**Yes**		**No**	

**By ticking this box, I agree to an anonymised report of this survey being published**	

**Please confirm that you are a patient in Ireland or Northern Ireland who has been diagnosed with prostate cancer.**	**Yes**		**No**	

**Please confirm that you have undergone surgery or radiotherapy or drug treatment, either on its own or in combination, to treat your prostate cancer.**	**Yes**		**No**	

**Please indicate how you will complete the survey?**	
• **Independently**	
• **With the help of a partner / relative / friend**	
• **A combination of independently and with the help of a partner / relative / friend**	

Together, we're finding answers to cancer.

Demographics

(For any question where you can type a response, please take care not to include any person's name or personal information for anonymity and privacy reasons)

**1. Age**	

**2. Year of prostate cancer diagnosis – please provide best estimate**	

**3. What is the highest level of education you have attained?**	
• No formal education	
• Primary school	
• Secondary School	
• Third level	

**4. County of Residence (required purely to estimate geographic representation)**							
Antrim		Armagh		Carlow		Cavan	
Clare		Cork		Derry		Donegal	
Down		Dublin		Fermanagh		Galway	
Kerry		Kildare		Kilkenny		Laois	
Leitrim		Limerick		Longford		Louth	
Mayo		Meath		Monaghan		Offaly	
Roscommon		Sligo		Tipperary		Tyrone	
Waterford		Westmeath		Wexford		Wicklow	

**5. What treatment have you had to date for your prostate cancer?** *(please tick all that apply)*	
• Surgery (Radical prostatectomy)	
• Radiotherapy	
• Brachytherapy	
• Hormone treatment (injections / tablets)	
• Chemotherapy	
• Focal therapy (Cryotherapy / High-intensity focused ultrasound)	
• Active surveillance or watchful waiting	
• I don't know / unsure	
• Other	
If other, please specify	

Together, we're finding answers to cancer.

**6. Have you finished your treatment?**	**Yes**		**No**	
**If yes, how long has it been since you finished? (Your best estimate)**	**Years and Months**			
**If No, what treatment are you currently receiving?**				
• Surgery (Radical prostatectomy)				
• Radiotherapy				
• Brachytherapy				
• Hormone treatment (injections / tablets)				
• Chemotherapy				
• Focal therapy (Cryotherapy / High-intensity focused ultrasound)				
• Active surveillance or watchful waiting				
• I don't know				
• Other				

**7. Which of the following best represents your sexual orientation?**	
• Asexual	
• Bisexual	
• Gay	
• Heterosexual or straight	
• Lesbian	
• Pansexual	
• Prefer not to say	

**8. Do you consider yourself to be transgender?**	**Yes**		**No**	

**9. Are you in a relationship at present that did (or still does) involve sexual activity?**	**Yes**		**No**	

**10. Would you consider yourself sexually active without a partner**	**Yes**		**No**	

**11. Would you consider your sexual health to be important at present?**	**Yes**		**No**	

Together, we're finding answers to cancer.

Persons understanding and knowledge of prostate cancer and the impact of treatment on sexual wellbeing.

(For any question where you can type a response, please take care not to include any person's name or personal information for anonymity and privacy reasons)

**12. Before diagnosis, I had a good knowledge and understanding of prostate cancer and the impact of treatment on sexual wellbeing**	
• Strongly Agree	
• Agree	
• Neither Agree nor disagree	
• Disagree	
• Strongly Disagree	

**At the time I started my treatment, I had a good knowledge and understanding of prostate cancer and the impact of treatment on sexual wellbeing**	
• Strongly Agree	
• Agree	
• Neither Agree nor disagree	
• Disagree	
• Strongly Disagree	

**Today, I have a good knowledge and understanding of prostate cancer and the impact of treatment on sexual wellbeing**	
• Strongly Agree	
• Agree	
• Neither Agree nor disagree	
• Disagree	
• Strongly Disagree	

Cancer Trials Ireland
Together, we're finding answers to cancer.

13. <u>**How prepared did you feel you were for the impact of treatment on sexual function?**</u>	
• Very prepared	
• Adequately prepared	
• Under prepared	
• Completely unprepared	
**If Under-prepared or Completely unprepared; what would have helped you prepare more?**	
• More or clearer written information	
• More or clearer discussion with my medical team / doctor	
• Other, please specify:	

**14. How would you rate the impact of treatment on your sexual wellbeing based on what you expected?**<br><br>**The impact has been:**	
• Much better than I expected	
• Better than I expected	
• What I expected	
• Worse than I expected	
• Much worse than I expected	

**15. Was there any discussion of the impact of prostate cancer treatment on sexual function with you in a healthcare setting?**	
• Yes	
• No	
• Unsure / I don't know	
• I don't recall discussion but I was given written information e.g. booklets / leaflets	
**If there was any discussion on the impact of prostate cancer treatment, who discussed the impact of prostate cancer treatment on sexual function with you?** *(Please tick all that apply)*	
• Urologist (surgeon)	
• Radiation Oncologist	
• Medical Oncologist	
• Specialist nurse	
• GP	
• I cannot remember / I don't know	
• Other	
If other, please specify	
**If yes, who initiated the discussion (brought up the subject) about the impact of treatment on sexual function?**	
• I did	
• The doctor – Urologist, Radiation Oncologist, Medical Oncologist	
• Nurse	
• GP	
• My partner	
• Unsure / I don't recall	
• Other	
If other, please specify	

cancer trials ireland

Together, we're finding answers to cancer.

**16. Thinking about the information on the effects of treatment on sexual function and wellbeing provided by your medical team / in a healthcare setting / from your GP / Nurse - What format was the information given in?** *(please tick all that apply)*	
• Verbal – spoken	
• Written – booklet, leaflet	
• Online link, website	
• Not applicable – I didn't receive ANY information	
• Other	
If other, please specify	
**How reliable / trusted did you consider the information at the time?**	
• Very reliable	
• Quite reliable	
• Neither reliable nor unreliable	
• Quite unreliable	
• Very unreliable	
**How clear / legible / understandable was the information provided?**	
• Very easy to understand	
• Quite easy to understand	
• Neither easy nor difficult to understand	
• Quite difficult to understand	
• Very difficult to understand	
**How personalised / tailored to you and your situation / circumstances was the information provided throughout your diagnosis and treatment?**	
• Very personalised / tailored to me and my situation	
• Quite personalised / tailored to me and my situation	
• Somewhat personalised / tailored to me and my situation	
• A little personalised / tailored to me and my situation	
• Not at all personalised / tailored to me and my situation	

Together, we're finding answers to cancer.

The following questions 17-25 relate to your initial consultations around the time of your diagnosis when your treatment options were discussed.					
**17.** Did you have a consultation with a Urologist? (Surgeon)	Yes		No		Unsure / Not known	

**18.** Did you have a consultation with a Radiation Oncologist? (Radiotherapy specialist)	Yes		No		Unsure / Not known	

**19.** Did you have a consultation with a Medical Oncologist? (Consultant who looks after drug treatments / chemotherapy)	Yes		No		Unsure / Not known	

**20.** Were you offered a consultation with another consultant in a different specialty (e.g., Urologist referral to a Radiation Oncologist or vice versa) to discuss alternative treatment options?	Yes		No		Unsure / Not known	

**21.** Were you offered a second consultation i.e., within the same specialist (e.g., Urologist to Urologist or Radiation Oncologist to Radiation Oncologist)	Yes		No		Unsure / Not known	

**22.** If yes to either of the above, did you undertake the consultation?	Yes		No		

**23.** Did you feel there was adequate time given to consider your treatment options?	
• Strongly agree	
• Agree	
• Neither agree nor disagree	
• Disagree	
• Strongly disagree	

**24.** Overall, did you have adequate opportunity / time at appointments to ask questions?	Yes		No	

**25. Were there important questions that you wanted to ask but for some reason didn't?**	**Yes**		**No**	
**If yes, why did you not ask these questions?** *(Please tick all that apply)*				
• **I was reluctant / did not wish to discuss the issues with my partner / family member present**				
• **I did not feel it was important to me**				
• **I was embarrassed / uncomfortable discussing sexual wellbeing**				
• **The topic did not come up / Doctor did not bring it up or the doctor / scenario did not invite questions**				
• **I was overwhelmed / distressed by my cancer diagnosis and did not consider beyond treatment**				
• **Other**				
If other, please specify				

cancer trials ireland
Together, we're finding answers to cancer.

Mental Health

(For any question where you can type a response, please take care not to include any person's name or personal information for anonymity and privacy reasons)

**26. Were mental health support services *within the hospital* offered to you at the time of your diagnosis?**	**Yes**	**No**	**Unsure / I don't recall**

**27. In your experience has your mental health been impacted since your prostate cancer diagnosis and / or treatment?**	**Yes**	**No**
**If yes, have you sought help / support from any of the following?** *(please tick all that apply)*		
Psychosocial Oncology service at your cancer centre		
Social worker		
Psychologist		
Psychiatrist		
Counselling service at Cancer Support Centre		
Counselling service outside of Cancer Support Centre		
My consultant / treating doctor		
GP		
Advanced nurse practitioner / specialist nurse		
Family / friends		
Yoga / Meditation / other holistic therapies		
Online / Websites (Irish Cancer Society, Movember, etc)		
I have not sought any help or support		
Other		
If other, please specify		

**28. Have you been prescribed medication for your mental health (low mood / anxiety) as a result of your diagnosis and / or treatment?**	**Yes**	
	**I was previously taking prescribed medication for mental health and the dosage remained unchanged**	
	**I was previously taking prescribed medication for mental health and the dosage was increased**	
	**No**	
	**Prefer not to say**	

cancer trials ireland

Together, we're finding answers to cancer.

**29. <u>Before diagnosis</u>, how would you rate your mental health?**	
Excellent	
Very good	
Good	
Fair	
Poor	

**<u>After diagnosis and before treatment</u>, how would you rate your mental health?**	
Excellent	
Very good	
Good	
Fair	
Poor	

**<u>On the day your treatment finished</u>, how would you rate your mental health?**	
Excellent	
Very good	
Good	
Fair	
Poor	
Not applicable – I'm still having treatment / my treatment hasn't started yet	

**<u>At your lowest point since your cancer diagnosis</u>, how would you rate your mental health?**	
Excellent	
Very good	
Good	
Fair	
Poor	

**<u>Today</u>, how would you rate your mental health?**	
Excellent	
Very good	
Good	
Fair	
Poor	

**30. To what extent have sexual wellbeing concerns or sexual function impacted your mental wellbeing?**	
• Very much	
• Quite a bit	
• Somewhat	
• A little bit	
• Not at all	

cancer trials ireland

Together, we're finding answers to cancer.

Sexual Wellbeing

(For any question where you can type a response, please take care not to include any person's name or personal information for anonymity and privacy reasons)

**31. How important was sexual activity to you before your cancer diagnosis?**	
• Very important	
• Fairly important	
• Neither important nor unimportant	
• Fairly unimportant	
• Very unimportant	

**32. How important is sexual activity to you now?**	
• Very important	
• Fairly important	
• Neither important nor unimportant	
• Fairly unimportant	
• Very unimportant	

Together, we're finding answers to cancer.

33. Have you experienced any of these side effects or changes to your sexual wellbeing since your treatment? *(Please tick all that apply)*	
Erection problems	
Changes in penis length / shape	
Ejaculation problems	
Leaking urine during sexual activity	
No orgasm	
Changes in orgasm	
Problems during anal sex (receiving)	
Loss of sex drive	
Loss of self-esteem	
Loss of sexual confidence	
Relationship problems	
Frustration at not being able to help to satisfy a partner's needs.	
Don't feel like a man – lack of confidence in one's own sexual appeal and / or 'manliness'	
Anxiety immediately prior to sexual activity	
Avoidance based on prior experience	
Overall sense of anxiety / dread	
Fatigue (Too tired for sexual activity)	
None of the above	
Other	
If other, please specify	

Together, we're finding answers to cancer.

**Of these side effects or changes to your sexual wellbeing, which <u>FIVE</u> have been the most bothersome ?** *(Please select the top 5 side effects or changes to your sexual wellbeing that you find the most bothersome. If you have experienced less than 5 side effects / changes, please select the relevant ones only. If "none of the above" please enter 1 in the relevant box)*	
Erection problems	
Changes in penis length / shape	
Ejaculation problems	
Leaking urine during sexual activity	
No orgasm	
Changes in orgasm	
Problems during anal sex (receiving)	
Loss of sex drive	
Loss of self-esteem	
Loss of sexual confidence	
Relationship problems	
Frustration at not being able to help to satisfy a partner's needs.	
Don't feel like a man – lack of confidence in one's own sexual appeal and / or 'manliness'	
Anxiety immediately prior to sexual activity	
Avoidance based on prior experience	
Overall sense of anxiety / dread	
Fatigue (Too tired for sexual activity)	
None of the above	
Other	
If other, please specify	

**34. Since treatment, have you experienced changes to your feeling of sexual wellbeing and satisfaction?**	**Yes**		**No**	
**(If yes), how much of a change have you experienced to your feeling of sexual wellbeing and satisfaction?**				
• **Very big change**				
• **Big change**				
• **Moderate change**				
• **Small change**				
• **Very small change**				
**If yes, have you sought help or support from your medical team (consultant / nurse / GP)?**	**Yes**		**No**	
**If no, why did you not seek help or support?** *(Please tick all the apply)*				
• I was reluctant / did not wish to discuss the issues with my partner / family member present				
• I did not feel it was important to me				
• I was embarrassed / uncomfortable discussing sexual wellbeing				
• The topic did not come up / Doctor did not bring it up or the doctor / scenario did not invite questions				
• There wasn't enough time at any doctor's appointment to ask questions				
• I was overwhelmed / distressed by my cancer diagnosis and did not consider beyond treatment				
• Other				
If other, please specify				
**If Yes, what supports were offered?** *(Please tick all that apply)*				
• Counselling / Psycho-Oncology / Psychologist				
• Referral to sex / couples therapy				
• Prescription for medication				
• Prescription for injections or topical agents				
• Referral to specialist erectile dysfunction clinic, e.g. vacuum pump clinic / injectable clinic				
• Online / Website links				
• No supports were offered				
• Other				
If other, please specify				
**If supports were offered, how satisfied were you with the supports offered overall**				
• Very satisfied				
• Satisfied				
• Neutral				

cancer trials ireland
Together, we're finding answers to cancer.

• Dissatisfied	
• Very dissatisfied	

Together, we're finding answers to cancer.

**35. How would you rate your <u>current</u> satisfaction with your sexual wellbeing?**	
• Very satisfied	
• Satisfied	
• Neutral	
• Dissatisfied	
• Very dissatisfied	

**36. Since treatment, have you experienced urinary incontinence?**	**Yes**		**No**	
**If yes, how much of a problem is this for you today?**				
• A big problem				
• A moderate problem				
• A small problem				
• A very small problem				
• No problem				
**If yes, is the urinary incontinence improving over time?**				
• Yes				
• No				
• Unsure				
**If yes, to what extent do you feel that your sex life has been affected by your urinary incontinence symptoms?**				
• Very much				
• Quite a bit				
• Somewhat				
• A little				
• Not at all				

**Were you offered assistance with / treatment for Urinary Incontinence?**	
• Yes	
• No	
**If no, would you like assistance with this problem?**	
• **Yes**	
• **No**	

cancer trials ireland

Together, we're finding answers to cancer.

**37. Since treatment, have you experienced obstructive urinary symptoms – difficulty passing water, painful urination, poor flow, urinating a lot at night etc?**	**Yes**		**No**	
**If yes, how much of a problem is this for you today?**				
• A big problem				
• A moderate problem				
• A small problem				
• A very small problem				
• No problem				
**If yes, are the obstructive urinary symptoms improving over time?**				
• Yes				
• No				
• Unsure				
**If yes, to what extent do you feel that your sex life has been affected by your obstructive urinary symptoms?**				
• Not at all				
• A little				
• Somewhat				
• A lot				

**Were you offered assistance with / treatment for obstructive Urinary symptoms?**
• Yes
• No
**If no, would you like assistance with this problem?**
• **Yes**
• **No**

cancer trials ireland

Together, we're finding answers to cancer.

**38. Since treatment, have you experienced bowel symptoms – pain, passing more wind than before, leakage from back passage, diarrhoea, constipation, bleeding etc?**	**Yes**	**No**
**If yes, how much of a problem is this for you today?**		
• A big problem		
• A moderate problem		
• A small problem		
• A very small problem		
• No problem		
**If yes, are the bowel symptoms improving over time?**		
• Yes		
• No		
• Unsure		
**If yes, to what extent do you feel that your sex life has been affected by your bowel symptoms?**		
• Not at all		
• A little		
• Somewhat		
• A lot		

**Were you offered assistance with / treatment for bowel symptoms?**	
• Yes	
• No	
**If no, would you like assistance with this problem?**	
• **Yes**	
• **No**	

cancer trials ireland
Together, we're finding answers to cancer.

**39. Since treatment, have you been able to reach orgasm?**	**Yes**	
	**No**	
	**I don't know / I have not tried**	
**If yes, has it felt different than before treatment?**	**Yes**	**No**
**If yes, what has changed?** *(please tick all that apply)*		
• It's now painful		
• Feeling of "anti-climax"		
• No ejaculate		
• Orgasm associate incontinence		
• Less pleasurable than before treatment		
• More pleasurable		
• Not worth the effort		
• Other		
If other, please specify		

**40. Since your treatment, have you had difficulty reaching orgasm when masturbating?**	**Yes**	
	**No**	
	**I don't know / I have not tried**	
**If yes, what do you feel are the barriers to reaching orgasm?** *(please tick all that apply)*		
• Difficulty maintaining erection		
• Absent sex drive / libido		
• Reduced sensation of pleasure		
• Takes too long or cannot reach climax		
• Frustration and / or anxiety		
• Anger		
• Inability to relax / Over-thinking it		
• Other		

Together, we're finding answers to cancer.

**41. Since your treatment, have you had difficulty reaching orgasm with a sexual partner?**	**Yes**	
	**No**	
	**I don't know / I have not tried**	

**If yes, what do you feel are the barriers to reaching orgasm?** *(please tick all that apply)*	
• Difficulty getting or maintaining erection	
• Absent sex drive / libido	
• Reduced sensation of pleasure	
• Takes too long or cannot reach climax	
• Embarrassment / Self-conscious	
• Frustration and / or anxiety	
• Inability to relax / Over-thinking it	
• Other	
If other, please specify	

**42. If unable to reach orgasm or experiencing unsatisfactory changes in orgasm after treatment, how would you describe the impact or feeling of loss this has had on you?**	
• A great deal of impact	
• A lot of impact	
• A moderate amount of impact	
• A little impact	
• No impact at all	
• Not applicable	

**43. Were you informed either by your consultant, nurse or GP that it is possible to masturbate (and achieve orgasm) with a flaccid i.e., non-erect penis?**	**Yes**	**No**

Together, we're finding answers to cancer.

**44. Have you tried any penile or erectile dysfunction rehabilitation methods after your treatment?**	**Yes**	
	**No**	
**If yes, what rehabilitation methods have you tried?** *(please tick all that apply)*		
• Talking to partner / family / close friend		
• Counselling / Psycho-Oncology / Psychologist		
• Erectile dysfunction clinic		
• Sex Therapist		
• Pelvic floor exercises		
• Lifestyle modifications, e.g. Stopping smoking or cutting down alcohol		
• Taking medications used to treat erection problems - PDE-5 Inhibitors, e.g. Cialis, Viagra, Levitra, Spedra		
• Vacuum pump		
• Creams – Vitaros		
• Penile Injection		
• Penile pellets		
• Penile implants		
• Other		
If other, please specify		
**(If yes) If you have tried any penile or erectile dysfunction rehabilitation after your treatment, did you find it helped?**		
• Extremely helpful		
• Very helpful		
• Somewhat helpful		
• Not very helpful		
• Not at all helpful		

cancer trials ireland

Together, we're finding answers to cancer.

**45. Have you used any of the following to improve sexual pleasure post treatment?** *(please tick all that apply)*	
• Sex toys or aids	
• Visual aids, e.g. pornography	
• Lubricants	
• Amyl-nitrate / recreational drugs	
• Vacuum devices	
• Exploring other non-penetrative ways of pleasure	
• None of the above	
• Other	
If other, please specify	
**If you have used any of the above to improve sexual pleasure post treatment, did you find it helped?**	
• Extremely helpful	
• Very helpful	
• Somewhat helpful	
• Not very helpful	
• Not at all helpful	
• Not applicable (Not applicable (can be removed if question doesn't appear if "None of the above" selected above)	

**46. Are you comfortable trying new ways to achieve sexual satisfaction outside of penetrative intercourse?**	
• Very much	
• Quite a bit	
• Somewhat	
• A little	
• Not at all	
• Not applicable	

Together, we're finding answers to cancer.

47. Are you satisfied with hugging, kissing and other intimate activities, rather than penetrative sexual intercourse?	
• Very much	
• Quite a bit	
• Somewhat	
• A little	
• Not at all	
• Not applicable	

48. Are you comfortable talking with your partner about the changes to your sex life after prostate cancer treatment?	
• Very much	
• Quite a bit	
• Somewhat	
• A little	
• Not at all	
• Not applicable	

49. Regarding your connection as a couple, your emotional relationship, have you experienced changes in your relationship with your partner since your prostate cancer diagnosis and treatment?	
• I have not noticed any changes	
• Positive changes noticed	
• Changes noticed – neither positive nor negative	
• Negative changes noticed	
• Not applicable	

cancer trials ireland

Together, we're finding answers to cancer.

**50. What do you miss most about sex before your treatment?** *(Please tick all that apply)*	
• Having a happy sex life	
• Normal ejaculate volume	
• Spontaneity	
• The original feeling of orgasm	
• An erection sufficient for penetrative intercourse (without using rehabilitation methods)	
• Sexual self-confidence	
• Feeling sexually attractive to your partner	
• Feeling virile / potent / manly	
• Sense of overall sexual pleasure	
• Sensation of pleasure at orgasm	
• Some change but I don't miss anything	
• Not applicable/No change, everything is the same/None of the above.	
• Other	
If other, please specify	

**Of these, which <u>THREE</u> do you miss the most?** *(Please select the top 3 things you miss the most)*	
• Having a happy sex life	
• Normal ejaculate volume	
• Spontaneity	
• The original feeling of orgasm	
• An erection sufficient for penetrative intercourse (without using rehabilitation methods)	
• Sexual self-confidence	
• Feeling sexually attractive to your partner	
• Feeling virile / potent / manly	
• Sense of overall sexual pleasure	
• Sensation of pleasure at orgasm	
• Some change but I don't miss anything	
• Not applicable/No change, everything is the same/None of the above.	
• Other	
If other, please specify	

**In the final section of this survey, we would like to ask your opinion on the topics of consent and Data Protection (GDPR) in clinical trials. These questions are not specifically related to the topic of this survey but relate more broadly to clinical research.**

**51. Consider the following scenario – you have been diagnosed with lung cancer, and your treating doctor has recommended that you participate in a clinical trial. After having all aspects of the trial and treatment explained to you by your medical team, you agree (consent) to take part in the trial.**<br><br>**In this scenario, if all your identifiable information was removed, would you be willing to extend consent to include use of your health information/samples for existing, or future research projects in any form of cancer?**	
• Yes	
• No	
• Unsure	

**52. The General Data Protection Regulation (GDPR) is an EU law that protects personal data and privacy for individuals within the EU, including Ireland. Under GDPR, data must be processed lawfully, fairly, and transparently, collected for specific purposes, minimized, accurate, stored only as necessary, and secured. Irish individuals have rights to access, rectify, erase, restrict processing, data portability, and object to data processing, including automated decision-making. Amongst other things, organizations in Ireland must ensure explicit consent for processing sensitive data.**<br><br>**Should the Government simplify how GDPR is applied in some areas and remove some restrictions in relation to use of health information for research purposes?**	
• Strongly agree	
• Agree	
• Neither agree nor disagree	
• Disagree	
• Strongly disagree	
